# Peer support during in vivo exposure homework to reverse attrition from prolonged exposure therapy for posttraumatic stress disorder (PTSD): description of a randomized controlled trial

**DOI:** 10.1186/s13063-020-04302-5

**Published:** 2020-04-28

**Authors:** Melba A. Hernandez-Tejada, Wendy Muzzy, Matthew Price, Stephanie Hamski, Stephanie Hart, Edna Foa, Ron Acierno

**Affiliations:** 1 Department of Psychiatry and Behavioral Sciences, Trauma and Resilience Center, McGovern Medical School at UTHealth, Houston, TX USA; 2 Ralph H. Johnson VA Medical Center, Charleston, SC USA; 3 Medical University of South Carolina College of Nursing, Charleston, SC USA; 4 Department of Psychiatry, University of Pennsylvania Perelman School of Medicine, Philadelphia, PA USA

**Keywords:** Peer support, Veterans, PTSD, Dropout, Prolonged exposure therapy

## Abstract

**Background:**

Effective treatments for posttraumatic stress disorder (PTSD) (e.g., prolonged exposure (PE); cognitive processing therapy (CPT)) exist and are widely adopted by the Departments of Veterans Affairs (VA) and Defense (DoD). Unfortunately, dropout from these treatments regularly exceeds 30%. However, in a recent survey of patients who dropped out of PE, approximately half indicated a greater likelihood of completion *if a peer who had completed treatment were available to help with the in vivo exposure homework.*

**Methods:**

We will use a between-groups randomized controlled design with repeated assessment at baseline, post treatment, and 3- and 6-month follow-up across measures of PTSD, depression, and functioning with 150 veterans who have indicated that they intend to drop out of treatment. Participants will be randomly assigned to one of two PE + Peer Support conditions: (1) a peer will offer support directly during in vivo exposure homework for 3–4 weeks; vs (2) a peer will call weekly for 3–4 weeks to offer general support and to check in on treatment progress.

**Discussion:**

The present study was designed to test the hypothesis that dropout from exposure-based PTSD treatment may be mitigated by using peers as support agents *directly* during PE in vivo homework experiences. Specifically, we intend to determine: whether patients who have dropped out of PE and are offered the “in vivo peer” adjunctive component to PE therapy will (1) return and complete treatment and (2) evince reduced PTSD symptomatology, compared to the same PE treatment, but with general peer support more reflective of current VA practices.

**Trial registration:**

This study protocol is approved and information is available at ClinicalTrials.gov, ID: NCT03485391. Registered on 2 April 2018.

## Introduction

### Background and rationale

Posttraumatic stress disorder (PTSD) is a debilitating mental health condition associated with diminished occupational and social functioning, poor physical health outcomes, substance abuse, depression, and risk of suicide [1–6]. Active-duty servicemen and women and veterans of the nation’s armed forces are at elevated risk of PTSD due to exposure to combat-related trauma, accidents, and other aspects of military service [7, 8]. The Institute of Medicine [9], the American Psychological Association [10] and Departments of Defense and Veterans Affairs (DoD, VA, respectively [11]) have identified cognitive behavioral therapies (CBTs), such as prolonged exposure (PE) [12] and cognitive processing therapy (CPT) [13], as those with the strongest evidence of their efficacy in treating PTSD. Human suffering and health service utilization by those affected by PTSD may be reduced if evidence-based treatments (EBTs), such as PE and CPT, were offered *and completed* [14–17].

Attrition from evidence-based therapy for PTSD is about 25–40%, even under optimum, research protocol settings [18–20]. Therefore, identifying and resolving barriers to effective treatment engagement and completion is essential. Our preliminary interview data from veterans who had dropped out of treatment indicate that *in vivo social support* in the form of having an accompanying partner who is another veteran (peer) may help non-adherent to treatment veterans to complete the most challenging parts of treatment [20]***.*** While novel in PTSD treatment, peer assistance has been used successfully by other health specialties (e.g., diabetes) [21] and may well be applied to PE.

Toward this end, we proposed pairing veterans who had successfully completed PE and no longer meet the criteria for PTSD, with veterans who are currently in treatment and at high risk of dropout or have dropped out from treatment. These peers would meet veterans at their in vivo exposure homework site to offer encouragement and support during the exposure homework. Veterans serving as peers (“workout buddies”) do not perform therapy; rather they provide support and encouragement.

### Hypotheses

#### Hypothesis 1

Treatment “dose,” measured in terms of number of sessions completed post randomization, will be greater for participants in the PE + Peer Support during the in vivo exposure assignment condition compared to the PE + Standard Supportive Peer condition.

#### Hypothesis 2

Treatment “outcome,” measured in terms of PTSD symptom reduction scores, will be significantly more improved for participants in the PE + Peer Support during the in vivo exposure assignment condition relative to the PE + Standard Supportive Peer condition at each assessment point (post treatment, 3-month and 6-month follow-ups).

### Trial design

Using a two-arm, randomized controlled trial design with repeated measures at baseline, post treatment, and 3- and 6-month follow-ups, we will compare the PE + Peer Support intervention to PE + Standard Supportive Peer to determine whether the former has greater impact on the following two primary endpoints: (1) treatment dose/completion and (2) treatment clinical outcome. The exploratory aims are to determine whether the program results in (3) differential treatment completion and outcomes for telemedicine vs in-person-delivered PE treatment, as preliminary data indicate that the peer support adjunctive component may be more relevant to veterans receiving PE via telemedicine, a treatment delivery format of increasing prevalence in VA and DoD settings.

(*Note*: (a) whereas telemedicine-delivered psychotherapy involves a therapist at one site and a patient at a second, all exposure therapy peers will be geographically located near patients and peer support exposure hierarchy homework assignments with participants take place in person, in the community. As we have done successfully in our feasibility study, special efforts will be made to recruit peers from across our service area so that they will be available for rural-residing veterans; (b) peers will not be conducting any therapy or substituting for the therapist, who will continue to deliver treatment. Rather, they will simply be meeting patients at in vivo, community homework assignment sites, such as the parking lot of a department store, and accompanying them during the exposure trial, which typically simply involves staying in that location until anxiety subsides.

## Methods: participants, interventions, and outcomes

### Study setting

Participants will be 150 veterans of the Operation Iraqi Freedom (OIF), Operation Enduring Freedom (OEF), Operation New Dawn (OND), Persian Gulf or Vietnam war theaters with PTSD, male or female, aged 18 years and above, who have dropped out of, or indicated that they intend to drop out of, evidence-based treatment for PTSD (PE). The recruitment site is the VAMC in Charleston, SC, USA and the Affiliated Clinic in Hinesville (Savannah, GA, USA). Research is conducted ethically in accordance with the World Medical Association Declaration of Helsinki.

### Eligibility criteria

Excluded from the study are those with active psychosis or a diagnosis of dementia, suicidal ideation or clear intent, and current substance dependence; however, to maximize generalization of results, the presence of mildly problematic substance use and other forms of psychopathology will not be a basis for exclusion. All structured interviews will be audiotaped to calculate inter-rater reliability on a randomly selected 20%. Based on clinic data and data from two prior DoD/VA PE treatment outcome studies, we will have satisfactory minority representation: 35–50% African American, 8–10% Hispanic, and 4–6% Asian American; 10% are expected to be women.

#### Medication stabilization

Patients meeting the inclusion criteria will be asked to maintain medications at current dosages where medically possible. Participants who have not initiated new prescription medications in the previous 4 weeks will initiate treatment 1 week following the assessment battery. However, potential participants who have recently begun trials of prescription medication will be required to wait 4 weeks post assessment to ensure medication stabilization, at which point the assessment battery will be re-administered. This re-administered battery will be used for pre-treatment data in analyses.

### Recruitment

We have obtained Institutional Review Board (IRB) and Veterans Affairs Research and Development (VA RD) approval to allow our clinical research staff to approach all veterans receiving services at our local VA who have dropped out of, or indicate that they intend to drop out of, PE with an invitation to participate in the peer support study. Related to the question of whether or not participants meeting the inclusion criteria are available, is whether or not those available participants will actually agree to return to treatment. Our pilot study data showed that over 50% of those who have dropped out of PE agree to return with the assistance of a peer, and 30% of these signed consents do so immediately.

In addition to direct contact with recent dropout patients from therapy provided by VA clinicians, we will post IRB-approved recruitment flyers in prominent locations in VA hospitals and clinics within our VAMC catchment area. The recruitment flyer will provide information about the study and a telephone number that interested subjects can call to receive detailed information. We will also list this study among those catalogued in our consortium institution’s “active research projects” web page and weekly newspaper research recruitment sections, two popular forums for recruiting subjects locally. Third, veterans for whom PTSD is suspected in the primary care visit and on general mental health visit may be referred to the study by these clinicians. Our final recruitment strategy will be to run a series of advertisements in relevant local newspapers. The advertisements will provide brief information about the study and a telephone number for interested participants to call.

#### Recruitment steps

Potential participants will be directly contacted by study staff when recruitment derives from the weekly PTSD clinic staff meetings. Potential participants who respond to brochures, or who are referred by other providers will be directed to the study coordinator for face-to-face explanation of the study in a private treatment room at the VAMC, where they will review informed consent and have all questions answered. All potential participants will first complete the assessment interview; immediately after the assessment interview, participants meeting inclusion and exclusion criteria (specified above) will be randomized to condition by the study coordinator using the randomization protocol designed by the study statistician. Participants will be told to expect a telephone call from their counselor to start treatment within 7 days.

#### Participant payments

All participants will receive US$30 for the baseline assessment, US$30 for the post-treatment assessment, US$35 for the 3-month follow-up assessment, and US$45 for the 6-month follow-up assessment for a combined possible total of US$140.

### Description of the informed consent process

Informed consent will be administered by approved individuals trained in human-subjects’ regulations and informed consent procedures, with appropriate VAMC and University training certifications on file and up to date. Informed consent will be collected at the study research offices where potential candidates will be invited to learn about the study. Informed consent will be collected in a private, interruption-free environment. Potential candidates will not be required to make a decision to participate at this initial contact, though that possibility will be available. However, if they wish to discuss participation with significant others, they will be encouraged to do so. If any potential volunteers are illiterate, the consent form will be verbally read and explained to the potential participant volunteer in the presence of a witness.

**Fig. 1:**
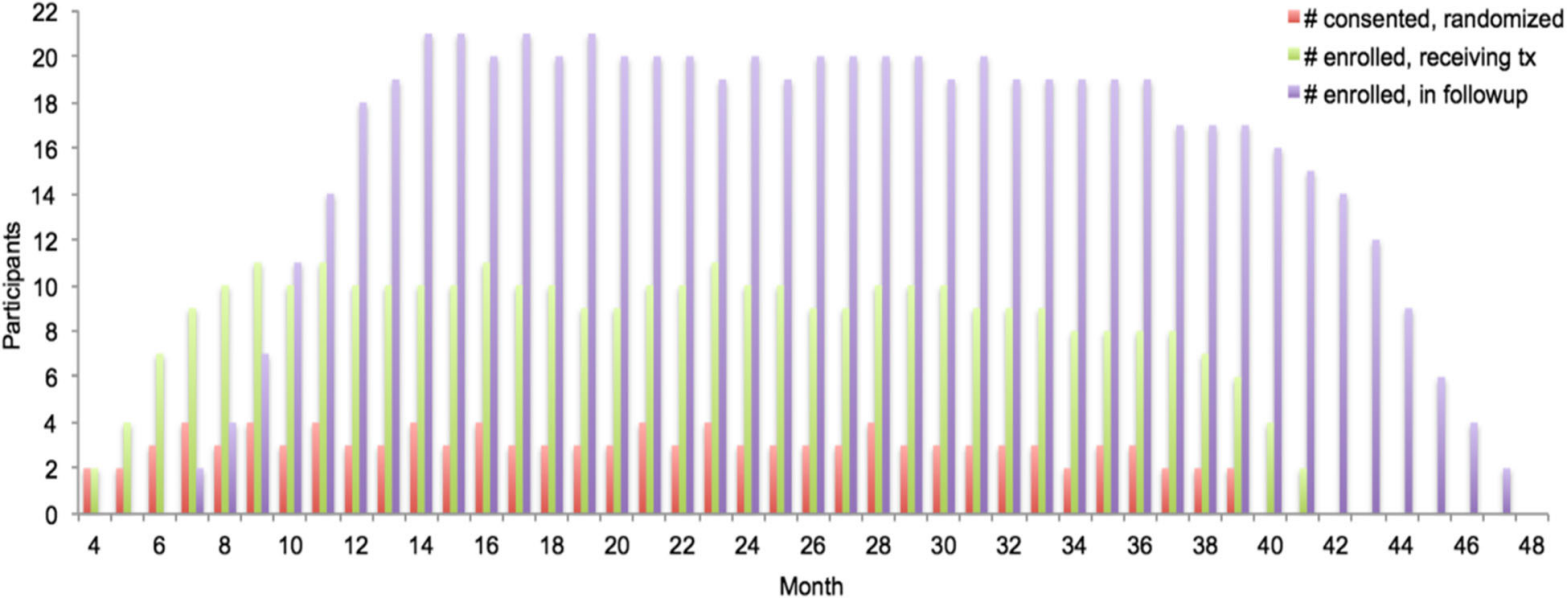
Recruitment and enrollment targets

Participants will be told that this is a study where there is a 50–50 chance of receiving PE with a peer during in vivo exposure; that is, a peer who has successfully been through the experience, and who will encourage and support them during in vivo (as opposed to imaginal) exposure trials, such as going to Wal-Mart, etc.), or receiving PE with a generally supportive peer, who will call them weekly to check in on how treatment is going. The call is brief and should not take more than 5 min to complete. Those individuals who indicate that they would like to try treatment again will continue PE treatment from the point of their last session. If more than 3 months has elapsed since their last session, they will begin from PE session 1 (Fig. 1).

### Randomization procedures

A block randomization schedule stratified by race (African American, Other) and gender will be generated. Participants will be randomly assigned (1:1) to one of the two study conditions. After determining eligibility, enrolled patients will be assigned to treatment groups by the project coordinator and research assistant using a web-based computer-generated randomization scheme through REDCap, an application designed to support data capture for research studies. Study personnel will be able to obtain randomization assignment by completing a REDCap survey verifying inclusion/exclusion criteria.

Randomization will occur at the patient level. Once a patient is randomized, they will be entered into the study and included in the intent-to-treat analysis plan. The only members of the research team who will be aware of randomization assignment will be the project therapists, the research assistant, and the statistical analyst in charge of randomization. Assessors will be blinded to the conditions.

### Attrition and retention

As this is a study on treatment dropout, study participants have already left PE treatment once and may arguably be more likely to do so again in the future. As such, this study has been powered to offset a projected 35% dropout rate during treatment, and a 10–15% loss to follow-up during the 6-month follow-up period. Patients who attend at least 10 sessions or evince two consecutive weeks with PTSD Clinical Checklist-5: Military Version (PCL-5) scores below the VA National Center for PTSD suggested cutoff of 33 [22] will not be considered dropout patients, and patients will be allowed to make up sessions where appropriate or necessary within a 4-week window. Make-up sessions for subjects in both conditions will be handled in the same manner: when feasible, these sessions will be scheduled for later the same week.

### Treatments

#### Peer selection

Both treatment conditions use peers to supplement PE treatment, and the same peers will be used in both conditions, thereby controlling for non-specific factors associated with individual peers. Contamination across conditions is rather easy to avoid given the nature of the peer activities in the two conditions: the experimental condition uses peers who provide support *directly during scheduled in vivo exposure homework* (see below) and the control condition uses peers in a manner consistent with current VA practices insofar as they offer frequent telephone support to “check in” to see how VA treatment is progressing and offer support to continue treatment, *but never engage in any support during in vivo exposure therapy homework*. VA PTSD clinic therapists will be asked if they have any patients who have completed treatment and did very well, both overall and in particular with in vivo exposure therapy components. Therapists will then be asked to contact these patients, inform them about the exposure therapy workout buddy program, and ask them if they would like a member of our VA/DoD research team to discuss the program further. Upon being contacted, the research team will outline the nature of expectations and research characteristics, and schedule a training session. Thus, initial nominations for the program are based on therapist impressions of successful candidates after they have completed treatment. As such, the therapists know the candidates’ strengths. Peers who are candidates will complete informed consent, and then be evaluated for the presence of a PTSD diagnosis via the Clinician Administered PTSD Scale-5 (CAPS-5), and only those who no longer have the PTSD diagnosis will be permitted to participate in this study. Peers will not be matched on gender or age, except in cases of Military Sexual Trauma, for which peers are matched on gender. Peers will be selected on the basis of geographic proximity to participants.

### Experimental condition

#### PE + Exposure Peer Support

The experimental treatment is a modified version of PE insofar as social support in the form of peer workout buddies for in vivo exposure homework is added to the protocol. Specifically, PE is a manualized treatment [12] based on emotional processing theory, which suggests that traumatic events are incompletely and inaccurately encoded in memory as “fear networks.” Gradual exposure to corrective information via the confrontation of (i.e., exposure to) conditioned traumatic stimuli within a safe and therapeutic environment results in a competing and antithetical memory structure that inhibits the conditioned fear response. PE relies on two primary therapeutic tools: in vivo exposure and imaginal exposure. During in vivo exposure, the patient confronts feared, but safe, stimuli or cues that elicit trauma-related distress. During imaginal exposure, patients “re-live” the traumatic event, providing a detailed verbal account that includes sensory information, thoughts, feelings, and reactions experienced during the traumatic event. PE includes the following session by session components: (1) psychological education about the common reactions to traumatic events and presentation of the treatment rationale (sessions 1 and 2), (2) repeated in vivo exposure to traumatic stimuli such as people, places, things, or situations that resemble or trigger memories of the traumatic event but are realistically safe (in vivo exercises are assigned as homework during sessions 3 through 11), (3) repeated, prolonged, imaginal exposure to traumatic memories (imaginal exposure is implemented during sessions 3–11; patients listen to session audiotapes for homework between sessions), and (4) relapse prevention strategies and further treatment planning (session 12).

In the spirit of the new VA mandate to use peer specialists in specialty clinics such as PTSD clinics, we will offer those individuals who indicate that they have decided to drop out or have dropped treatment (e.g., stopped attending sessions) the opportunity to have a peer (see description of peer and training below) who has been through treatment successfully and no longer meets PTSD diagnosis help them to complete in vivo exposure trials as a peer, by offering social support and encouragement during exposure trials. Specifically, participants who have dropped out from treatment will be asked if they would like to try PE treatment again, this time with a peer for support.

#### Exposure peer support assignment training

*Note:* the major activities of the peers are to meet veterans at in vivo exposure homework sights and offer encouragement and support. Thus, logistics and limits of responsibility, not skills, are the primary focus of training. Time is spent emphasizing appropriate boundaries and safety procedures. Each peer will attend a 4-h training meeting. During this meeting, the rationale for in vivo exposure will be reviewed, and the benefits of having a supportive partner, friend, or workout buddy during in vivo exposure will be outlined. Training will explicitly include content wherein peers will be clearly informed that they are not engaging in the role of therapist or providing therapy; rather, their role is equivalent to that of a group member in traditional supportive counseling, only this support is given in the context of exposure exercises. In such a situation, group members offer each other advice and support on how to achieve the stated goals. In the present case, the stated and agreed-upon goal will be the in vivo exposure therapy assignment. Peers in both conditions will be trained in the importance of confidentiality, and are not paid or compensated for their time. Limits to personal responsibility will be clearly outlined, and peers will be educated that neither the outcome of the treatment, nor the disposition of the patient is their responsibility. We will review potential negative outcomes, and apply the limits of personal peer responsibility to each. Moreover, we will make it explicitly clear that exactly, and only, those roles and responsibilities found in supportive counseling group sessions, including support, respect, and confidentiality, are in place here, with the exception that meeting locations are offsite and without therapist direction.

#### Exposure homework logistics

Our pilot study experience allowed us to refine the methods of initiating the peer support to the following steps: once patients have agreed to re-initiate treatment, peers will be instructed by the project coordinator to call in via televideo/meet in person to the next therapy session to make introductions and listen to the patient and therapists review the next item of the in vivo exposure hierarchy in depth. The location, timing, outline, description, and parameters of the in vivo exposure therapy assignment will be reviewed and will be clear to patient, peer, and therapist. Once this clarity has been achieved, peer and patient finalize arrangements to meet at a set time and place to engage in this exposure trial and will be directed to arrange three such meetings per week for 3–4 weeks. The analogy of a workout buddy will be used, and the purposely time-limited aspect (one quarter of the normal treatment length) of peer support for in vivo trials will be restated.

For patients who dropped out of treatment prior to developing and engaging in hierarchy item exposure, the peer support “session call-in” will be delayed until this hierarchy is established. Once established and reviewed, the peer will call into the next session and discuss the logistics of meeting the patient at the item location with patient and therapist. Upon re-entry to treatment, participants, peers, and therapists will hold a conference call to review in vivo exposure trials with which exposure homework peer support will assist and a brief discussion of logistics will be held. Then, therapists will speak with only the participant to assure that they continue to want to participate with a peer. The therapists will ask the patients to comment on any problems or benefits associated with in vivo exposures accompanied by the peer during treatment sessions each subsequent week to obtain a progress report of how the exposure trials are going, and to determine whether there were any issues that should be discussed in person with the participant. After the first peer exposure event, therapists will also contact the peer to determine whether they would like to continue with the participant and if there are any issues or problems. If a peer indicates that they would no longer prefer to work with a particular participant, a different peer will be assigned. If two peers indicate for any one participant that they prefer not to work with that participant, the peer support will be terminated for that participant, and therapists will include these interpersonal events as topics of therapeutic focus.

#### In vivo exercises with peers

All peers engage only in hierarchy items jointly determined by the therapist and participant, with assent of the peer. No new, or unscheduled exposure activities, initiated on the part of the peer or the participant are permitted. Peers are directed to communicate with participants if either party feels or seems to feel uncomfortable with the activity in question. The difference between discomfort arising from conditioned anxiety that is part of PTSD and discomfort arising from feelings that the situation is inappropriate are discussed with the therapists when needed.

Only situational activities, in clearly safe places, will be included in the in vivo exposure participation events by peers. This will be determined by review with the therapist and the peer. If either party feels that there is more than minimal risk, the activity will not be included. Note, the risk of anxiety on the part of the participant is not a reason to exclude an activity (indeed, producing and dealing with such anxiety in a supportive environment is the point of in vivo exercises). It is impossible to list every possible activity that should be avoided vs included. However, several common activities are precluded. Specifically, we will exclude:

- Activities involving driving together on the part of either the peer or the peer as driver (public transportation, such as taking a bus together is permitted)
- Activities in or around private residences of the participant or peer
- Activities involving weapons (e.g., shooting range)
- New or previously unlisted or unscheduled activities; and
- Activities that are associated with risk or danger as defined per therapist and or peer

Peers and participants are told that the peer support is only in place for a given patient for 3–4 weeks, three to four times per week, and is a means by which to allow participants to progress to their own, independently conducted in vivo exposure trials. Thus, “phase out” of the program is built in from the beginning. Peers will focus on helping participants to engage in those exposure exercises that typically are social in nature. Again, any exposure trials, such as driving, that require the participant to engage alone, or from which peers are specifically prohibited will not be the target of this program. If participants and workout buddies agree, the length of time for exposure trial assistance can be extended to 6 weeks, three to four times per week, but this will be based on therapist judgment with respect to therapeutic gains vs risk of becoming dependent on workout buddy.

### Control condition

#### PE + Standard Peer Support

Participants in this condition will receive PE (see Foa, Hembree, & Rothbaum, 2007 [12]) and will be assigned a peer volunteer who will *not* engage in any support *during* the in vivo exposure homework. The primary purpose of the peer in this condition is to emulate standard VA PTSD peer support procedures. Therefore, these peers provide general support, such as reminding and encouraging participants to attend appointments, expressing concern when therapy appointments are missed, checking up on them once per week via telephone and asking about current treatment progress, life stresses, opportunities, problems and successes, and offering general support with VA programs. The PE component of this treatment will be matched to the experimental condition in terms of session number and duration (12 sessions); treatment completion will be defined identically across both conditions (minimum 10 sessions or pre-specified reductions in PCL-5 score at two consecutive sessions). Counselors will deliver treatment according to the same schedule as the experimental condition, once weekly for 75–90 min. Thus, the level of peer and patient contact in the standard peer support condition is designed to be the same as that of the experimental condition. Thus, peers call several times per week for 3–4 weeks at about sessions 3–8, offering support and encouragement.

### Treatment integrity and adherence

The present study will obtain a quantitative measure of protocol adherence through a checklist of specific procedures scheduled to be followed in the treatments outlined above. In order to ensure that treatments are competently administered in accord with the protocols as written, all sessions will be audiotaped, and 20% of these will be rated for competence and adherence by the co-investigators. This will also allow us to study any differences between conditions on specific and non-specific factors, such as length of therapy time, therapist empathy and rapport. Two raters, blind to treatment condition, will rate the tapes independently to allow for the computation of inter-rater reliability.

### Dependent measures

#### Interviewers

Each assessment interview will be conducted by raters blind to treatment condition. Raters will be qualified to master’s level and specifically trained in diagnostic interviewing techniques using the CAPS-5. Raters will be required to complete five practice standardized interviews and achieve item-level inter-rater reliability of at least 90%.

#### Clinical outcomes

The following interview and self-report measures have been widely used in the clinical evaluation of adults with PTSD, and will be used in the present study. They will be collected at baseline, post treatment, and 3- and 6-month follow-ups. The PCL-5 [22] and Beck Depression Inventory-II (BDI-II) [23] are also collected every 2 weeks during active treatment.

#### Clinician Administered PTSD Scale-5 (CAPS-5) [22]

PTSD diagnoses will be ascertained using the CAPS-5, considered the gold standard in PTSD assessment. The CAPS-5 is a 30-item, structured interview that corresponds to the *Diagnostic and Statistical Manual of Mental Disorders* (DSM-5) criteria for PTSD. For each diagnostic item, standardized questions and probes are provided. Questions focus on symptom presence, the onset and duration of symptoms, subjective distress, impact of symptoms on social and occupational functioning, improvement in symptoms since a previous CAPS administration, overall response validity, and overall PTSD severity. Psychometric validation is ongoing.

#### Structured Clinical Interview for DSM-5 (SCID-5) [24]

Major Depression, Panic, and Substance Dependence diagnoses will be evaluated using this structured clinical interview based on the DSM-5. The onset of each symptom set will be specified. With the previous version, Ventura and colleagues [25] found excellent inter-rater reliability across a variety of disorders (overall kappa = 0.85).

#### PTSD Clinical Checklist-5 (PCL-5) [22, 26]

The PCL-5 is a new version of the PCL, among the most commonly used self-report measures of PTSD symptom presence and intensity. Like its predecessor, the PCL-5 is structured to correspond to the DSM-5 PTSD criteria. The 20 items are scored on a 0–4 Likert scale for each symptom corresponding to “Not at all” to “Extremely.” Total scores on the PCL-5 range from 0 to 80. Initial psychometric data are encouraging. With college student samples, Blevin et al. found that PCL-5 scores exhibited strong internal consistency (*α* = .94), test-retest reliability (*r* = .82), and convergent (*rs* = .74 to .85) and discriminant (*rs* = .31 to .60) validity. With veteran samples, Bovin et al. (2015) found that the PCL-5 had good internal consistency (*α* = .96) and test-retest reliability (*r* = .84). Moreover, signal detection analyses using the CAPS-5 indicated a diagnostic cutoff score of 31–33 on the PCL-5 optimally categorized PTSD diagnosis.

#### Selected scales of the Deployment Risk and Resiliency Inventory (DRRI) [27]

The DRRI is collection of self-report measures assessing 14 key deployment-related risk and resilience factors with demonstrated implications for veterans’ long-term health.

#### Beck Depression Inventory-II (BDI) [23]

The BDI-II is a 21-item self-report scale, is among the most widely used instruments to measure depression. Beck et al. [28] demonstrated that the BDI-II has high internal consistency (*α* = .86–.91).

#### Health-related functioning and social support: Medical Outcome Study Short Form-36 Health Survey (SF-36) [28]

The SF-36 is a 36-item questionnaire that measures health status, social support, and functioning over the past 4 weeks. The SF-36 has good test-retest reliability as well as sensitivity to change in health status [29, 30]. Lin and Ward [31] found the SF-36 to have high internal consistency (Cronbach’s alpha > .87) and found that the subscale’s reliability coefficients ranged from .59 to .89.

### Process outcomes

During data collection of clinical outcomes, we will also examine the following measures immediately post treatment, and at 3- and 6-month follow-ups (only treatment credibility will be assessed at pre-treatment, after treatment has been explained):

#### Session attendance, session frequency limits, definition of treatment completion, and study attrition

This is, of course, a primary outcome variable related to aims 1a and 1b of this study, and examines the impact of the workout buddy program on “treatment dose” delivered, measured both in terms of total new treatment sessions attended and percentage completing treatment in each group. To this end, we will keep careful records of session attendance and study dropout. A treatment dropout patient will be defined as any patient who does not complete a total of at least 10 new sessions of treatment *or* who leaves treatment prior to a reduction of PCL-5 scores below the clinical cutoff posted by the VA National Center for PTSD of 33 obtained in two consecutive PCL-5 scores. A treatment completer will be defined as completing 10 or more sessions; or when no additional treatment is needed due to treatment gains, these gains will be reflected by two consecutive PCL-5 scores below 33. The overall limit to number of sessions in either group will be 15 (so as to prevent outliers in session frequency affecting dose between group analyses). It is expected that most patients will occasionally miss a session or fail to complete their homework assignments. These will not be considered dropout patients; patients will be allowed to make up sessions as long as they re-enter treatment within a 4-week period. Patients who miss all four treatment sessions during a 4-week period will be considered dropout patients (i.e., if a patient, during treatment, does not attend the next session within 4 weeks of the last session completed, they will be considered a dropout patient).

#### Treatment adherence

Treatments in both groups will require patients to carry out “homework assignments.” These forms will be collected and monitored each week during treatment. The percentage of returned, completed forms will be computed as an indicator of adherence to homework assignments.

#### Charleston Psychiatric Outpatient Satisfaction Scale (CPOSS-VA) [32]

Patient satisfaction is an important treatment outcome variable, closely associated with clinical outcomes [33]. The CPOSS-VA is 16-item measure, with a Likert scale response format, based on a general measure of patient satisfaction [34]. In a sample of veterans, preliminary data showed excellent reliability (alpha = .96) and good convergent validity with anchors (“would you recommend this treatment?”) [32].

### Statistical methods

#### Analysis plan for objective 1

We will adopt a relatively direct analytic approach for this first objective. Specifically, if preliminary descriptive analyses reveal baseline differences in psychopathology or other potentially confounding variables, including number of sessions completed prior to dropout (i.e., prior to entry into this study) these will serve as covariates in analysis of covariance (ANCOVA), which will be used to compare the average dose received in terms of number of treatment sessions in each group. These ANCOVAs will be repeated with race and gender as between-group factors to determine whether any interaction is present between treatment condition and these demographic characteristics. A similarly direct approach will be taken for the dichotomous variable of treatment completion, for which we will initially use chi-square analyses for bivariate examination of treatment completion in terms of each demographic variable and each treatment condition, followed by logistic regression in which those variables producing significant chi-square relationships to this outcome will be included in regression models. We will use 95% confidence intervals (CIs) for single proportions to estimate the proportion of participants who complete treatment within each intervention group. We will also estimate the difference in completer proportions between groups using 95% CIs for difference in proportions. In addition, frequency distributions describing participants’ reasons for discontinuation of study participation will be developed. In this way, the effect of each treatment condition on dose measured in terms of overall number of sessions completed, and in terms of proportion of participants completing at least 10 sessions/achieving clinical cutoff will be described.

#### Analysis plan for objective 2

Our primary outcome is the PCL-5 continuous score for PTSD. Clinical (CAPS-5, PCL-5, BDI-II), functional (SF-36), and process (treatment credibility scales, patient satisfaction, session rating scale, service delivery perceptions, retention in treatment) outcomes will be compared using the generalized linear mixed models (GLMM) approach.

### Data monitoring

Permanent monitoring of the study is performed to ensure that the study is following all procedures in the protocol. The monitoring occurs through weekly meetings, and monthly internal audits. The study personnel makes sure that case report forms and binders are up to date and timely reports of any adverse events or any other potential risk/harm happen within 24 h of first reporting.

## Discussion

The present study seeks to address the problem of dropout from evidence-based treatment for PTSD by examining the relative effectiveness of (1) supplementing evidence-based PTSD treatment with peer support during community based in vivo exposure homework from a peer (who has successfully completed treatment) (PE + In Vivo Exposure Peer Support) *vs*; (2) PE + Standard Peer Support (peers offer telephone-based support and in-person meetings at the VA to encourage treatment attendance, as is standard in VA peer support programs) in terms of process outcomes (i.e., session attendance) and clinical outcomes (i.e., symptomatology). Therefore, we chose a repeated-measures, randomized, between-groups design (*Treatment:* PE + In Vivo Exposure Peer Support vs PE + Standard Peer Support *by Time*: baseline, post treatment, 3-month and 6-month follow-ups).

Importantly, our pilot feasibility data support this intervention [20]. Fully 52% (*n* = 43) of 82 dropout patients in the feasibility study indicated that they “would be interested in trying exposure therapy for PTSD a second time if they could do so with the assistance of an exposure peer,” and 30% [13] of these *immediately* completed informed consent to re-initiate treatment. Given the strong support from veterans who had dropped out of treatment for the proposed peer support solution, we next tested the assumption that we could recruit peers to volunteer for such a role and attempted to identify peers who might be good candidates to serve in this capacity (e.g., offer to meet patients at in vivo homework locations to offer support and encouragement). Thirteen potential “exposure peers” were contacted and 11 agreed to enroll in the program as a volunteer, and completed complete training with the principal investigator (PI), indicating that recruitment of peers does not appear at all problematic. Note, each peer agreed to accompany up to four different patients over a 6-month period.

**Fig. 2:**
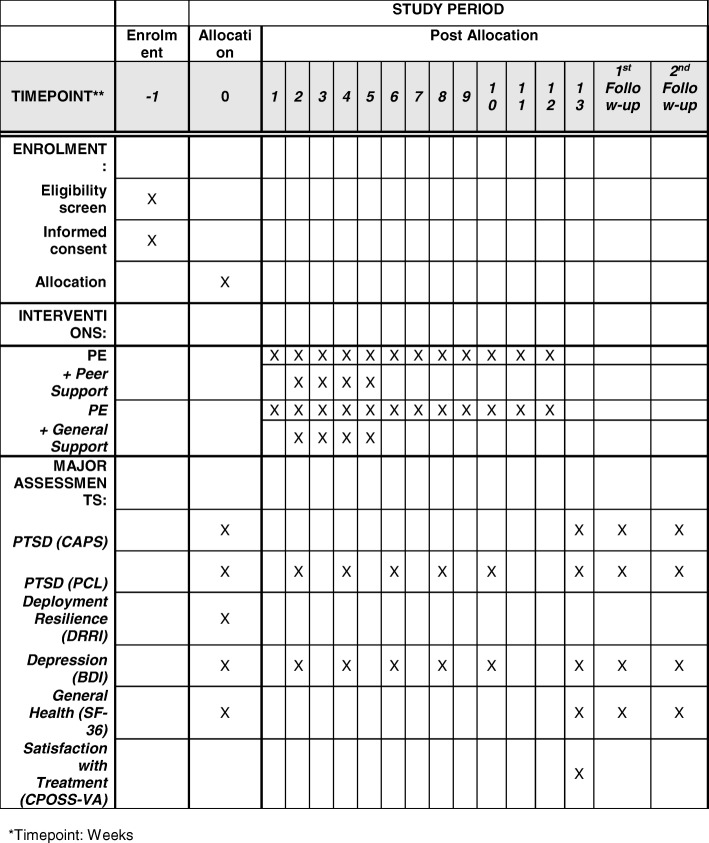
Time schedule of enrollment, interventions, and assessments. *Timepoint: weeks

Veterans’ suggestion to combine the beneficial aspects of social support found in group treatment with the effectiveness of exposure-based treatments for PTSD represented no less than a “Eureka” moment for our research team, insofar as we recognized that combining peer support with exposure therapy might directly impact, in a cost-effective manner, the treatment retention issue confronting patients of VA and DoD. This technique is directly in line with the recent DoD and Veterans Administration implementation of “Peer Support Programs” [35] across the country, is designed to emulate the peer support found in group exposure therapy for PTSD [36]. We predict that this support will help to address high dropout associated with exposure therapy, particularly when delivered via telemedicine, a modality of increasing prevalence across VA/DoD (Fig. 2).

### Trial status

Protocol version number: 0.17, date 8 October 2019. Protocol amendments have been submitted. Currently, the study is in the amendment #10. Recruitment began in September 2018. Recruitment completion will be in September 2022.

## Data Availability

Please contact the author for data requests.

## Abbreviations

ANCOVA: Analysis of covariance
BDI: Beck Depression Inventory
CAPS: Clinician Administered PTSD Scale-5
CI: Confidence interval
CPOSS: Charleston Psychiatric Outpatient Satisfaction Scale
CPT: Cognitive processing therapy
DoD: Department of Defense
DRRI: Deployment Risk and Resiliency Inventory
DSM: *Diagnostic and Statistical Manual of Mental Disorders*
GLMM: Generalized linear mixed models
IRB: Institutional Review Board
OIF: Operation Iraqi Freedom
OEF: Operation Enduring Freedom
OND: Operation New Dawn
PCL-5: PTSD Clinical Checklist-5: Military Version
PE: Prolonged exposure therapy
PTSD: Posttraumatic stress disorder
SCID: Structured Clinical Interview for DSM-5
SF: Short Form
VA: Veterans Affairs
VAMC: Veterans Affairs Medical Center
VA RD: Veterans Affairs Research and Development
